# Menopause and Depressive Symptoms in the UK Biobank

**DOI:** 10.1192/bjo.2025.10175

**Published:** 2025-06-20

**Authors:** Hira Mayet, Katie Marwick, Magdalena Pfaff, James M Stone

**Affiliations:** 1 Division of Psychiatry, University of Edinburgh, Edinburgh, United Kingdom; 2 NHS Lothian, Edinburgh, United Kingdom; 3 Department of Clinical Neuroscience, Brighton and Sussex Medical School, University of Brighton and University of Sussex, Brighton, United Kingdom; 4 Sussex Partnership NHS Foundation Trust, Worthing, United Kingdom

## Abstract

**Aims:** The UK Biobank is a large-scale prospective cohort study with data collected on over 500,000 individuals within the United Kingdom. We sought to understand whether the years around the menopause are associated with an increased likelihood of depressive symptoms, using the PHQ-2 questionnaire, which screens for depressive disorders. A score of 3 or above is suggestive of a depressive disorder. Our analysis looked at the likelihood of having significant depressive symptoms in the years before and after the final menstrual period.

**Methods:** Exclusion criteria included male participants, participants who had bilateral oophorectomies, those who were unsure of when their FMP was due to having a hysterectomy, those whose age at final menstrual period was under 40 or preferred not to say, and those whose reported age at final menstrual period differed by >2 years when asked on different occasions. Participants who did not give an answer to either of the two screening questions were also excluded.

The PHQ-2 enquires about depressive mood and anhedonia over the prior two weeks at baseline assessment. Possible answers were not at all, several days, more than half the days, and nearly every day. Using logistic regression, odds ratios were calculated for likelihood of having a PHQ-2 score above 3 from 9 years prior to the final menstrual period to 9 years after, compared with the year of the final menstrual period.

**Results:** In females undergoing natural menopause (n=143,685) those assessed who were within a year of their final menstrual period had the highest rate of depressive symptoms. 7.6% of women within a year of their final menstrual period had a PHQ-2 score of 3 or more. Depressive symptoms at all other timepoints were less frequent, ranging from 3.9% to 7.1%, with the OR compared with year of final menstrual period ranging between 0.49 to 0.93. In the 2 years either side of the final menstrual period, depressive symptoms were not significantly lower than the year of the final menstrual period, particularly at 2 years prior (6.0%, OR 0.77 (95% CI (0.58, 1.02)), one year post (6.9%, OR 0.90 (95% CI (0.78, 1.05)) and 2 years post (7.1%, OR 0.93 (95% CI (0.80, 1.08)).

**Conclusion:** These results suggest that the proportion of women experiencing significant depressive symptoms increases in the years around the final menstrual period. There is an increased likelihood of significant depressive symptoms in the year of the final menstrual period compared with surrounding years.

